# Otolaryngologists in facial plastic surgery, yes

**DOI:** 10.1590/S1808-86942011000400001

**Published:** 2015-10-19

**Authors:** José Eduardo Lutaif Dolci

**Affiliations:** MD. Otorhinolaryngologist. Chairman of the Brazilian Association of Otorhinolaryngology and Neck and Facial Surgery (ABORL-CCF), Full Professor and Director of the Graduate Program in Medicine – School of Medical Sciences - Santa Casa de São Paulo

Borders have always separated men, leading to greater or lesser conflicts. Not the imaginary lines of maps or landmarks in the cities of neighboring countries, but in the defense of geographic territories.

In Medicine, for good or for worse, things are not different. Plastic surgeons advocate that they are the only ones who can perform cosmetic surgeries. They complain of “invaders” from other specialties in their field of work.

In Brazil, as well as in other South and North American countries, physicians also perform plastic surgeries associated with their specialties. Ophthalmologists, for instance, they not only perform eye enucleation, but they also correct fallen eyelids. Mastology experts, after tumor resection, insert silicone prosthesis in the breasts of patients.

Who knows more about eyes than ophthalmologists? Otorhinolaryngologists are the ones who master the knowledge about the face. Therefore, why couldn't we do facial plastic or repair surgery?

This was our stand in a recent meeting we had with officials from the Brazilian Medical Association (AMB) and Federal Board of Medicine (CFM). Contrary to the sayings from some plastic surgeons, I believe there is a market for all of us, and the more professionals we have able to perform cosmetic surgery, for repair purposes or not, the better for patients and physicians alike.

With the greater longevity and consequent aging of the Brazilian population, people crave for, besides health, good appearance. It is a natural desire of human beings. Therefore, we need more doctors trained in this type of surgery. Including, of course, otorhinolaryngologists.

Another issue to consider is the chaotic Brazilian traffic which, unfortunately, is full of accidents. Traumas happen frequently; and we are called to treat the wounded, with repair and cosmetic surgeries in our specialty.

I do not agree that we should be prohibited from doing, for instance, a purely cosmetic rhinoplasty, when we are called to do a functional rhinoplasty. We, otorhinolaryngologists, are the better indicated physicians to perform these procedures, and other face-related ones. If we consider the degree of difficulty of ear surgeries, such as facial nerve decompression, which is performed by the otorhinolaryngologist, why not do an otoplasty (correction of protruded ear), which degree of difficulty is considerably smaller?Similar principle can be applied to a parathyroidectomy, when compared to a face-lift.

This does not mean an invasion from other areas, but an effort to provide the patient with a thorough treatment, with ethics, respect and professionalism.

The Sun, and plastic surgery, are not a privilege of some, but a right belonging to all of us.

